# Feeding styles and adiposity in children of 6 months– 5 years of age: Protocol for a systematic review and meta- analysis

**DOI:** 10.1371/journal.pone.0292139

**Published:** 2023-10-05

**Authors:** Divya Nair Haridas, Onno C. P. van Schayck, Giridhar R. Babu, N. Sreekumaran Nair, Prafulla Shriyan

**Affiliations:** 1 Department of Public Health Science, Indian Institute of Public Health Gandhinagar, Gandhinagar, Gujarat, India; 2 Public Health Foundation of India, New Delhi, India; 3 Department of Family Medicine, University of Maastricht, Maastricht, Netherlands; 4 Department of Epidemiology, Indian Institute of Public Health Bangalore, Bangalore, Karnataka, India; 5 Department of Biostatistics, Jawaharlal Institute of Postgraduate Medical Education & Research, Puducherry, India; University of Liege: Universite de Liege, BELGIUM

## Abstract

Obesity in children is a major public health concern due to the increased risk of developing adverse health outcomes in their future, and disability in adulthood. The existing systematic reviews on the topic are limited in scope, focusing solely on high-income countries and children aged 4–12 years. Hence, we propose to conduct a systematic review and meta-analysis to understand, how exposure to authoritative feeding style versus authoritarian, indulgent, uninvolved compare in terms of its association with adiposity in children aged 6 months to 5 years. Preferred Reporting Items for Systematic Review and Meta-Analysis Protocols (PRISMA-P) guidelines were followed for ensuring the completeness of the protocol. Case-control and cohort studies will be included. Searches will be done using electronic databases viz. PubMed, Ovid EMBASE, PsycINFO and Web of Science. Grey literature will be searched using OpenGrey and Grey Literature Report. We will only include quantitative studies using the developed search strategy. For categorical outcomes, relative risks, odds ratios, and hazard ratios with confidence intervals and for continuous outcomes mean difference with confidence intervals will be used. Risk of Bias In Non-randomized Studies- of Exposure (ROBINS-E) will be used for the evaluation of risk of bias in the individual observational studies. Considering the inherent variability in the observational studies, random effects meta-analysis will also be conducted. If between-study heterogeneity exists, a subgroup analysis based on low and middle-income countries vs. high income countries will be conducted. If the data is not suitable for combining quantitatively, a narrative synthesis will be undertaken. We propose to identify publication bias by using contour-enhanced funnel plots and “trim and fill” method. Outcome reporting bias will be ascertained by comparing the outcomes published in the protocol and the published report. The Grades of Recommendation, Assessment, Development, and Evaluation (GRADE) system will be used to understand the confidence we can have on the effect estimates.

**Registration:** This protocol has been registered in International Prospective Register of Systematic Reviews (PROSPERO) on 13 March 2023 with registration number CRD42023356014.

## Introduction

The accumulation of abnormal or excessive body fat that impairs health is defined as overweight and obesity. In children aged 5–19 years, a Body Mass Index-for-age (BMI-for-age) greater than two standard deviations above the World Health Organisation (WHO) growth reference median is considered obese [1]. In 2016, there were over 340 million children and adolescents aged 5–19 who were overweight or obese. This is a major public health concern given that obese children have an increased risk of developing adverse health outcomes in their future, and disability in adulthood [1]. The long-term consequences of childhood obesity are not limited to physical health, as it can also have a significant impact on a child’s emotional, social, and mental well-being [2]. As per the WHO, in 2020, 39 million children under the age of 5 were overweight or obese worldwide [1].

Understanding feeding styles is important in the field of child nutrition. Feeding styles are determined by the combination of two domains: demandingness and responsiveness. Demandingness refers to the degree to which a parent encourages their child to eat, while responsiveness refers to the way in which the parent encourages the child to eat, whether in a responsive or non-responsive manner. Feeding styles can be broadly categorized into four types: authoritative, authoritarian, indulgent, and uninvolved, based on the level of demandingness and responsiveness demonstrated by parents. The authoritative feeding style is characterized by high levels of both demandingness and responsiveness, while the authoritarian style involves high demandingness and low responsiveness. The indulgent feeding style is characterized by low demandingness and high responsiveness, whereas the uninvolved style involves both low demandingness and responsiveness [3].

Understanding the different feeding styles and their impact on child nutrition is crucial for developing effective interventions aimed at promoting healthy eating habits in children. Research in this area has implications for parents, caregivers, health professionals, and policymakers, who can use this knowledge to promote healthier eating practices and reduce the risk of childhood obesity and related health issues.

A review by Vollmer *et al*. found that while two studies did not show evidence of an association between feeding style and child weight, while five studies did suggest a significant association [4]. However, the existing systematic reviews on the topic are limited in scope, with one focused solely on high-income countries and the other specifically on children aged 4–12 years [5, 6]. Therefore, there is a need for a more comprehensive review of the literature to understand the association between feeding styles and adiposity in children between the ages of 6 months to 5 years. To address this gap, we propose to conduct a systematic review of all available published and unpublished literature on this topic. The review will aim to provide a clear understanding of the association between feeding styles and adiposity in young children, and whether this association is consistent across different income levels and in various public health contexts.

This systematic review will contribute to a better understanding of the impact of feeding styles on child adiposity and inform the development of evidence-based interventions to promote healthy eating habits in young children. Ultimately, this will help prevent childhood obesity and promote better health outcomes for children, families, and communities. This information can be vital for policymakers, parents, and healthcare providers to make informed decisions about the most effective feeding styles to prevent childhood obesity.

## Methods

PRISMA-P guidelines were followed, and PRISMA-P checklist was used for ensuring the completeness of the protocol [7].

### Objectives

#### Research question

In children aged 6 months to 5 years, how does exposure to authoritative feeding style versus authoritarian, indulgent, uninvolved compare in terms of its association with adiposity?

The objectives are,

1. To evaluate the association between the four feeding styles (authoritative, authoritarian, indulgent, uninvolved) and adiposity measures in children aged 6 months to 5 years.
2. To synthesize the existing evidence to present a comprehensive understanding of the relationship between the four feeding styles (authoritative, authoritarian, indulgent, uninvolved) and adiposity measures in children aged 6 months to 5 years.

We also aim to examine if the association differs across high-income countries and low- and middle-income countries.

### Eligibility criteria

#### Study designs

Observational studies such as case-control and cohort studies will be included. We are not expecting any experimental study in the study context as it may be unethical to force the parents to follow a particular feeding style which may have an ill effect on child-health in the long run.

#### Participants

Studies done on children who are in the age group of 6 months to 5 years and are not having adiposity/obesity or underweight before 6 months of age. Infants below 6 months and children above 5 years of age or having adiposity or overweight before 6 months of age will be excluded.

#### Exposure

Feeding styles and parenting styles categorised as authoritative, authoritarian, indulgent and uninvolved will be studied. Authoritative feeding style will be considered as the exposure. Studies describing feeding practices such as restriction, pressure to eat, rewarding etc. will be excluded.

#### Comparators

All other categories such as authoritarian, indulgent and uninvolved will be considered as comparators.

#### Outcome

Adiposity/Obesity in children of 6 months– 5 years age group will be included. Studies which reported outcomes such as, BMI, BMI z score, skinfold thickness, waist to hip ratio, overweight, weight for length\height, body fat percentage, fat mass index, total fat, regional fat, waist circumference, body weight, weight gain and weight loss will also be included. Measures other than the above mentioned will be excluded.

#### Setting

There will not be any restriction on the type of setting.

#### Language

Literature available in English language will be included for the systematic review. Literature available in any other language other than English will be excluded as there is evidence suggesting no systematic bias or impact on the effect estimates and conclusions by restricting the systematic review to English language literature in conventional medicine [8, 9].

#### Information sources

Searches will be done using electronic databases viz. PubMed, Ovid EMBASE, PsycINFO and Web of Science. Grey literature will be searched using OpenGrey and Grey Literature Report. Review of references and co-cited articles of the selected studies will be conducted, and domain experts will be contacted to identify any potentially missing references.

#### Search strategy

Quantitative studies alone will be searched using the search strategy developed. Studies published after 1900 will only be included by using the year of publication filter. The search strategy for PubMed is provided in S1 Appendix. This has been validated by checking whether all the major studies have been found by running the search.

#### Study records

EndNote 20 will be used to import and manage the articles. DNH and PS will independently search for the articles by using the search strategy developed. These articles will then be screened by title and abstract to check for their eligibility in Rayyan. A full text screening will be done for these articles for final inclusion. Disagreements between the two reviewers in any stage of screening will be resolved through discussion. DNH and PS will independently extract data by using the extraction form developed [10]. Disagreement, if any between the two reviewers will be resolved through discussion.

#### Data extraction

From the selected articles, data will be extracted on the study design, population including the sample size, number of events, baseline characteristics, outcome including definition and measurement, exposure variable and other predictors with the help of a predesigned data extraction proforma. Data will be extracted by DNH and checked for accuracy by PS. Any discrepancies will be resolved by discussion. Statistical data analyses results of unadjusted and adjusted effect estimates with standard errors including missing data will be extracted.

### Outcomes and prioritisation

#### Primary outcome

The primary outcome will be adiposity, for which measures such as adiposity, obesity, Body Mass Index, Body Mass Index z score, skinfold thickness, waist to hip ratio, weight for length\height, body fat percentage, fat mass index, total fat, regional fat, waist circumference and body weight will be considered.

#### Risk of bias in individual studies

Risk of Bias In Non-randomized Studies- of Exposure (ROBINS-E) will be used for the evaluation of risk of bias in the individual observational studies [11]. DNH and PS will evaluate the risk of bias in the studies. The studies identified as having high risk of bias will be used as a sub-group against moderate and low risk studies, if conducting a meta-analysis is deemed appropriate. Any disagreement between the reviewers will be solved by discussion.

#### Data synthesis

A meta-analysis by study designs will be conducted if the data is found appropriate for quantitative analysis. Considering the inherent variability in the observational studies, random effects meta-analysis will be conducted. Fixed effects meta-analysis will also be done on the same data as a part of sensitivity analysis. For adiposity/obesity outcomes, relative risks, odds ratios and hazard ratios (all covariate adjusted) with confidence intervals will be used. For continuous outcomes such as BMI, BMI z score etc., mean difference and confidence intervals will be used. Standardized mean differences and confidence intervals will be used if different measurement scales are used in the selected studies. Attempt will be made to obtain the missing data by contacting the authors. Cochrane handbook will be followed for methods to deal with missing data such as replacing median with missing mean, imputing mean from lower quartile, median and upper quartile measures, computing missing standard errors from confidence intervals or p values and missing standard deviations from standard error, confidence interval, t statistic or p value for mean difference. In the absence of the above-mentioned measures, interquartile range or range will be considered for the calculation of standard deviation. If none of the measures are available for computation, missing data will be imputed from the other available studies [12]. χ2 test for the Q statistic and *I*^2^ will be calculated for determining the heterogeneity between the studies. ‘One-out’ sensitivity analyses will be performed to identify the sources of heterogeneity by excluding one study at a time [13, 14] and a subgroup analysis based on Low and Middle-Income countries vs. high income countries will be performed. R 4.2.2 software will be used for the data analysis. If the data is not suitable for combining quantitatively, a narrative synthesis will be undertaken.

#### Meta-biases

Contour-enhanced funnel plots will be used to identify the publication bias [15]. The “trim and fill” method will be used to identify as well as correct the publication bias [16, 17]. Outcome reporting bias will be ascertained by comparing the outcomes published in the protocol and the published report. If the study protocol is unavailable, outcomes reported in the methods and results sections of the published report will be compared [7].

#### Confidence in cumulative estimates

The Grades of Recommendation, Assessment, Development, and Evaluation (GRADE) system will be used to understand the confidence we can have on the effect estimates. Domains assessed for quality of evidence will be study design, risk of bias, degree of inconsistency, imprecision, indirectness of results and reporting bias [7].

An independent quality rating will be done by DNH and PS. Disagreement between the two reviewers in the quality rating will be resolved by discussion.

## Discussion

In this protocol we have included the step-by-step process which will be followed for the planned systematic review. As per our knowledge there are no systematic reviews conducted on feeding styles and adiposity among children in the age as early as 6 months. Hence, this systematic review will be an exploration to understand whether there exists an association between feeding styles and adiposity in children. We find it important to study the 6 months-5 years age group as the complementary feeding starts at the age of 6 months and by 1 year the babies are introduced to every food an adult in the family consumes. The findings will help parents and public health experts to decide whether they should really focus on the feeding style to improve the child’s health and prevent adiposity. If there exists enough evidence for the association between feeding style and adiposity, parents may very well follow the feeding style appropriate for their children from the time feeding starts.

## Supporting information

S1 Checklist PRISMA-P 2015 Checklist. (PDF)

S1 Appendix PubMed search strategy. (PDF)

## References

[1] Obesity and overweight [Internet]: World Health Organization; 2021 [cited 2023 February 12]. Available from: https://www.who.int/news-room/fact-sheets/detail/obesity-and-overweight.

[2] Sahoo K, Sahoo B, Choudhury AK, Sofi NY, Kumar R, Bhadoria AS. Childhood obesity: causes and consequences. J Family Med Prim Care. 2015;4:187–192. doi: 10.4103/2249-4863.154628 25949965 PMC4408699

[3] Hughes SO, Power TG, Fisher JO, Mueller S, Nicklas TA. Revisiting a neglected construct: parenting styles in a child-feeding context. Appetite. 2005;44:83–92. doi: 10.1016/j.appet.2004.08.007 15604035

[4] Vollmer RL, Mobley AR. Parenting styles, feeding styles, and their influence on child obesogenic behaviors and body weight. A review. Appetite. 2013;71:232–241. doi: 10.1016/j.appet.2013.08.015 24001395

[5] Hurley KM, Cross MB, Hughes SO. A systematic review of responsive feeding and child obesity in high-income countries. J Nutr. 2011; 141:495–501. doi: 10.3945/jn.110.130047 21270360 PMC3040906

[6] Shloim N, Edelson LR, Martin N, Hetherington MM. Parenting Styles, Feeding Styles, Feeding Practices, and Weight Status in 4–12 Year-Old Children: A Systematic Review of the Literature. Front Psychol. 2015;6:1849. doi: 10.3389/fpsyg.2015.01849 26696920 PMC4677105

[7] Shamseer L, Moher D, Clarke M, Ghersi D, Liberati A, Petticrew M, et al. Preferred reporting items for systematic review and meta-analysis protocols (PRISMA-P) 2015: elaboration and explanation. BMJ. 2015 349:g7647. Erratum in: BMJ. 2016;354:i4068. doi: 10.1136/bmj.g7647 25555855

[8] Dobrescu AI, Nussbaumer-Streit B, Klerings I, Wagner G, Persad E, Sommer I, et al. Restricting evidence syntheses of interventions to English-language publications is a viable methodological shortcut for most medical topics: a systematic review. J Clin Epidemiol. 2021;137:209–217. doi: 10.1016/j.jclinepi.2021.04.012 33933579

[9] Morrison A, Polisena J, Husereau D, Moulton K, Clark M, Fiander M, et al. The effect of English-language restriction on systematic review-based meta-analyses: a systematic review of empirical studies. Int J Technol Assess Health Care. 2012;28:138–144. doi: 10.1017/S0266462312000086 22559755

[10] Dekkers OM, Vandenbroucke JP, Cevallos M, Renehan AG, Altman DG, Egger M. COSMOS-E: Guidance on conducting systematic reviews and meta-analyses of observational studies of etiology. PLoS Med. 2019;16:e1002742. doi: 10.1371/journal.pmed.1002742 30789892 PMC6383865

[11] Morgan RL, Thayer KA, Santesso N, Holloway AC, Blain R, Eftim SE, et al. A risk of bias instrument for non-randomized studies of exposures: a user’s guide to its application in the context of GRADE. Environ Int. 2019; 122: 168–184. doi: 10.1016/j.envint.2018.11.004 30473382 PMC8221004

[12] Higgins JP, Li T, Deeks JJ, editors. Chapter 6: Choosing effect measures and computing estimates of effect. 2022. In: Higgins JPT, Thomas J, Chandler J, Cumpston M, Li T, Page MJ, Welch VA (editors). Cochrane Handbook for Systematic Reviews of Interventions [Internet]. Cochrane. Available from: https://training.cochrane.org/handbook/current/chapter-06.

[13] Patsopoulos NA, Evangelou E, Ioannidis JP. Sensitivity of between-study heterogeneity in meta-analysis: proposed metrics and empirical evaluation. International journal of epidemiology. 2008;37:1148–1157. doi: 10.1093/ije/dyn065 18424475 PMC6281381

[14] Sutton AJ, Abrams KR, Jones DR, Sheldon TA, Song F. Methods for Meta-analysis in Medical Research. Chichester: WILEY; 2000.

[15] Page MJ, Higgins JP, Sterne JA. Chapter 13: Assessing risk of bias due to missing results in a synthesis. 2022. In: Higgins JPT, Thomas J, Chandler J, Cumpston M, Li T, Page MJ, Welch VA (editors) Cochrane Handbook for Systematic Reviews of Interventions [Internet]. Cochrane. Available from: https://training.cochrane.org/handbook/current/chapter-13.

[16] Duval S, Tweedie R. Practical estimates of the effect of publication bias in meta- analysis. Australas epidemiol. 1998; 5:14–17.

[17] Duval S, Tweedie R. Trim and fill: A simple funnel-plot-based method of testing and adjusting for publication bias in meta-analysis. Biometrics. 2000;56:455–463. doi: 10.1111/j.0006-341x.2000.00455.x 10877304

