# Correction: Feeding styles and adiposity in children of 6 months– 5 years of age: Protocol for a systematic review and meta- analysis

**DOI:** 10.1371/journal.pone.0322591

**Published:** 2025-04-11

**Authors:** Divya Nair Haridas, Onno C. P. van Schayck, Giridhar R. Babu, N. Sreekumaran Nair, Prafulla Shriyan

An additional affiliation is missing for the third author. Giridhar R. Babu’s is also affiliated with the Department of Population Medicine, College of Medicine, QU Health, Qatar University.
