# Supplementary material for: Feeding styles and adiposity in children of 6 months– 5 years of age: Protocol for a systematic review and meta- analysis
Source: PLoS One. 2023 Oct 5;18(10):e0292139. doi: 10.1371/journal.pone.0292139 (PMC10553248; doi:10.1371/journal.pone.0292139)
Supplement: S1 Appendix — (PDF) [file pone.0292139.s002.pdf]

PubMed search strategy

(Pediatric Obesity[mesh] OR ((infant[mesh] OR "child, preschool"[mesh] OR child*[tiab] OR preschool*[tiab] OR "pre-school"[tiab] OR infant*[tiab] OR toddler*[tiab] OR kindergart*[tiab] OR pediatri*[tiab] OR paediatri*[tiab] OR boy[tiab] OR boys[tiab] OR girl*[tiab]) AND (obesity[mesh] OR "Body composition"[mesh] OR obes*[tiab] OR "body mass index"[tiab] OR BMI[tiab] OR adipos*[tiab] OR "body fat"[tiab] OR overweight[tiab] OR weight*[tiab] OR "body composition*" [tiab]))) AND (parenting[mesh] OR ((parents[mesh] OR parent*[tiab] OR carer*[tiab] OR caregiver*[tiab] OR mother*[tiab] OR maternal[tiab] OR father*[tiab] OR paternal[tiab]) AND (Authoritarianism[mesh] OR Permissiveness[mesh] OR style*[tiab] OR indulgent[tiab] OR permissive[tiab] OR authoritative[tiab] OR authoritarian[tiab] OR uninvolved[tiab] OR neglectful[tiab])))
